# Gut microbiota dysbiosis and altered short-chain fatty acid biosynthetic pathways in Chinese children with obesity and nonalcoholic fatty liver disease

**DOI:** 10.3389/fendo.2026.1965476

**Published:** 2026-10-30

**Authors:** Xin Yuan, Ying Zhang, Xiangquan Lin, Ruimin Chen

**Affiliations:** Department of Pediatric Endocrinology, Genetics and Metabolism, Fuzhou First General Hospital Affiliated with Fujian Medical University, Fuzhou Children’s Hospital of Fujian Medical University, Fuzhou, China

**Keywords:** 16S rRNA, ANGPTL4, Bacteroides, children, gut microbiota, nonalcoholic fatty liver disease, short-chain fatty acids

## Abstract

**Background:**

Nonalcoholic fatty liver disease (NAFLD), recently reclassified as metabolic dysfunction-associated steatotic liver disease (MASLD), is the most prevalent chronic liver disease in children, affecting 13% of the general pediatric population and up to 47% of children with obesity worldwide. Gut microbiota dysbiosis contributes to NAFLD pathogenesis through the gut-liver axis, yet the interplay between microbial communities and host metabolic regulators, particularly angiopoietin-like protein 4 (ANGPTL4), remains incompletely characterized.

**Methods:**

Eighty-nine Chinese children aged 5–15 years were enrolled, including 29 healthy controls (Con), 39 with simple obesity (OB), and 21 with obesity and NAFLD. Gut microbiota was profiled by 16S rRNA gene sequencing (V3–V4 region) and analyzed using QIIME2 and MicrobiomeAnalyst. Functional prediction employed PICRUSt2 referencing MetaCyc pathways. Serum ANGPTL4, leptin, adiponectin, and interleukin-6 were measured.

**Results:**

Chao1 species richness differed significantly among groups (F = 4.98, p = 0.009), while Shannon diversity did not (F = 2.22, p = 0.115). Beta diversity (PERMANOVA, F = 2.87, R^2^ = 0.063, p = 0.002) differed significantly among groups, although PERMDISP indicated heterogeneous dispersion (F = 6.62, p = 0.002). LEfSe analysis identified enrichment of short-chain fatty acid (SCFA)-producing genera (Bacteroides, Alistipes, Oscillospira, Parabacteroides) in healthy controls, while Megamonas, Blautia, Coprococcus, and Dorea characterized the NAFLD group. ANCOM-BC2 identified significant enrichment of Megamonas in the NAFLD group (q = 0.005 vs Con). PICRUSt2 predicted lower abundance of nine SCFA-related pathways in the obesity groups compared with controls. After FDR correction, Bacteroides-ANGPTL4 was the most significant correlation (r = 0.460, q < 0.001), and four additional associations survived correction. In hierarchical regression, Bacteroides remained independently associated with ANGPTL4 after adjustment for age, BMI, sex, and Tanner stage (β = 0.254, p = 0.016, ΔR^2^ = 0.057), although this association was attenuated after additional adjustment for group status (β = 0.204, p = 0.058).

**Conclusion:**

Chinese children with obesity and NAFLD exhibit gut microbial dysbiosis characterized by reduced species richness, depletion of SCFA-producing genera, and lower predicted abundance of SCFA-related pathways. The independent association between Bacteroides and ANGPTL4 suggests a potential microbiota-host metabolic link that may be associated with hepatic lipid metabolism.

## Introduction

Nonalcoholic fatty liver disease (NAFLD), recently reclassified as metabolic dysfunction-associated steatotic liver disease (MASLD) (1), has become the most prevalent chronic liver disease in children and adolescents worldwide. A recent systematic review estimated the global prevalence of pediatric NAFLD at 13% in the general population and 47% among children with obesity (2), with an increasing disease burden documented globally (3, 4).

The gut microbiota has emerged as a critical regulator of host metabolism through the gut-liver axis (5). Alterations in gut microbiota composition and function have been consistently linked to NAFLD in both adult and pediatric populations (6–8). A recent multi-omics study in Chinese children with MASLD identified distinct gut microbiota signatures and metabolite profiles (6), while a meta-analysis of shotgun metagenomic sequencing demonstrated that gut microbial composition is progressively altered with disease severity from obesity to steatohepatitis (7). A comprehensive review of pediatric MASLD and the gut microbiome emphasized that microbiota-derived metabolites including short-chain fatty acids (SCFAs), bile acids, and ethanol contribute to disease pathogenesis, though current research is constrained by small sample sizes and methodological heterogeneity (8).

SCFAs, primarily acetate, propionate, and butyrate, are produced through microbial fermentation of dietary fiber and play essential roles in maintaining intestinal barrier integrity, regulating hepatic lipid metabolism, and modulating systemic inflammation (9, 10). In NAFLD, the depletion of SCFA-producing bacteria and lower predicted abundance of SCFA-related pathways have been documented (11, 12). However, the majority of studies have been conducted in adults, and data in children remain limited.

Angiopoietin-like 4 (ANGPTL4), also termed angiopoietin-related protein 4 (ARP4) or fasting-induced adipose factor (FIAF), is a circulating inhibitor of lipoprotein lipase (LPL) that plays a central role in plasma triglyceride regulation (13). Colonization of germ-free mice with gut microbiota suppresses intestinal Angptl4 expression, increasing LPL activity and promoting fat storage, establishing ANGPTL4 as a key mediator of microbiota-driven metabolic regulation (14). In our previous work, serum ANGPTL4 levels correlated positively with specific gut bacterial genera in obese Chinese children (15). A recent review of Bacteroides in NAFLD highlighted its protective role through improvement of insulin resistance and enhancement of intestinal barrier function (16). However, the microbiota-ANGPTL4 axis in pediatric NAFLD remains unexplored.

In this study, we aimed to: (1) characterize the gut microbiota composition and predicted functional capacity in Chinese children with simple obesity and obesity with NAFLD compared with healthy controls; (2) examine associations between differentially abundant genera and serum metabolic markers; and (3) evaluate whether specific taxa are independently associated with serum ANGPTL4 after adjustment for confounders.

## Methods

### Study population

Participants were recruited from the Department of Endocrinology, Genetics and Metabolism of Fuzhou Children’s Hospital of Fujian Medical University between September 2017 and March 2018. Inclusion criteria were: (a) age between 5 and 15 years, and (b) residence in Fujian province. Exclusion criteria included: any endocrine disorder; history of antibiotic therapy within 3 months prior to enrollment; chronic gastrointestinal illness or use of gastrointestinal-related medication; diarrheal disease (per World Health Organization definition) within 1 month prior to enrollment; or hospitalization (>24 h) within 6 months prior to enrollment.

The participants were drawn from a previously established cohort of Chinese children with obesity, which has been analyzed in relation to insulin resistance (15), glucose metabolism (17), hyperuricemia (18), and metabolic status (19). The present study is a secondary analysis with distinct objectives: inclusion of a NAFLD group, PICRUSt2-based functional prediction, and examination of the Bacteroides-ANGPTL4 association. Clinical, anthropometric, and sequencing data were generated at recruitment (2017–2018); all functional and association analyses are newly reported.

Based on weight status and the presence of NAFLD, participants were divided into three groups: healthy normal-weight controls (Con), children with simple obesity without NAFLD (OB), and children with obesity and NAFLD (NAFLD). Participants with body fat percentage (BF%) ≥ 30% were diagnosed with obesity (20, 21).

NAFLD was diagnosed by abdominal ultrasonography performed by an experienced pediatric radiologist who was blinded to the participants’ laboratory and metabolic status. Diagnosis was based on the presence of increased liver echogenicity (bright liver) compared with the renal cortex, with or without hepatomegaly, in accordance with established pediatric criteria (22). Steatosis severity was not graded. Alternative causes were excluded by hepatitis B serology (for ALT > 2× ULN), medical history (chronic disease, medications, smoking, alcohol), and blood count/physical examination (acute infection). Recruitment predated the MASLD nomenclature; NAFLD is used throughout.

### Clinical assessment

Height and weight were measured to the nearest 0.1 cm and 0.1 kg, respectively, using a calibrated stadiometer and digital scale, with participants wearing light clothing and no shoes. BMI was calculated as weight (kg) divided by height squared (m^2^). BMI z-scores were calculated based on Chinese reference values (23). Waist circumference was measured at the midpoint between the lower border of the ribs and the iliac crest at the end of normal expiration using a non-elastic tape to the nearest 0.5 cm. Tanner staging was assessed by a pediatric endocrinologist and recorded as an ordinal score from 1 to 5, which was treated as a continuous variable in regression models.

### Laboratory measurements

After a 12-hour overnight fast, venous blood was collected. FPG, TC, TG, HDL-C, LDL-C, ALT, AST, and UA were measured by standard enzymatic methods (Beckman Coulter AU5800). Fasting insulin was determined by chemiluminescent immunoassay (IMMULITE 2000, Siemens). Body fat percentage was measured by bioelectrical impedance analysis (Inbody J20). Serum leptin, ANGPTL4 (ab99974), IL-6, and adiponectin were measured using commercial ELISA kits (Abcam, Cambridge, UK). The ANGPTL4 assay had a detection range of 27.4–20,000 pg/mL.

### Fecal sample collection, DNA extraction, and 16S rRNA sequencing

Fecal samples were collected on the day of enrollment and stored at −80 °C. Microbial DNA was extracted using the QIAamp DNA Stool Mini Kit (Qiagen) with bead-beating. The V3–V4 region of the 16S rRNA gene was amplified and sequenced on the Illumina MiSeq platform (BGI, Shenzhen, China), generating 2 × 300 bp paired-end reads. Total sequencing depth was 1,162,890 reads, with a mean of 13,066 reads per sample (range: 5,676–24,689).

### Bioinformatics analysis

Raw sequencing data were processed using QIIME2 (version 2019.7) (24) with DADA2 (25) for denoising, dereplication, and chimera removal, generating amplicon sequence variants (ASVs). Taxonomic classification was performed using a Naive Bayes classifier trained on the Greengenes 13_8 99% reference database (26). After denoising and filtering, 141 ASVs were retained from 154 original features.

Downstream analyses were conducted in MicrobiomeAnalyst (27). Filtering was performed on the ASV table after taxonomic assignment, using a minimum count of 4 in at least 20% of samples (low-count filter) and removal of features in the lowest 10% by interquartile range (low-variance filter). Rarefaction was not performed; instead, the filtered ASV table was normalized by total sum scaling to account for differences in sequencing depth. No samples were excluded during bioinformatic processing.

Alpha diversity (Shannon, Chao1) was compared by Welch’s ANOVA with *post-hoc* pairwise comparisons, as Levene’s test indicated unequal variances among groups. Beta diversity (Bray-Curtis) was assessed by PERMANOVA (999 permutations) and visualized by PCoA. PERMDISP (999 permutations) was used to test homogeneity of multivariate dispersions. LEfSe identified differentially abundant genera (LDA > 2.0) (28). To account for compositional effects and potential confounders, ANCOM-BC2 was additionally performed with adjustment for age, sex, and BF%.

Functional prediction used PICRUSt2 with MetaCyc pathways (29). Fourteen pathways related to SCFA biosynthesis and central carbon metabolism were prespecified based on prior literature, and group differences were tested by Kruskal-Wallis with Benjamini-Hochberg FDR correction.

### Statistical analysis

Clinical data were analyzed in SPSS 23.0. Normality was assessed by Kolmogorov-Smirnov test. Normally distributed variables were compared by one-way ANOVA with LSD *post-hoc* test; non-normally distributed variables by Kruskal-Wallis with Dunn-Bonferroni correction; categorical variables by chi-square or Fisher’s exact test.

Sample sizes were: 89 enrolled; 87 for regression (2 missing ANGPTL4); 85 for Spearman correlation (additional missing leptin/ALT); 84 for sensitivity analysis (2 additional missing ALT, excluding one extreme value). Alpha diversity, beta diversity, LEfSe, and PICRUSt2 included all 89 participants.

Spearman correlations were assessed with Benjamini-Hochberg FDR correction. Partial correlation and hierarchical linear regression adjusting for age, BMI, sex, Tanner stage, and group status evaluated the association of Bacteroides with ANGPTL4. ANCOM-BC2 verified differential abundance with adjustment for age, sex, and BF%. Two-tailed P < 0.05 was considered significant.

Regression diagnostics were performed for the hierarchical linear regression models. Residual plots were inspected, and the variance inflation factor was < 2 for all predictors, indicating no multicollinearity. ANGPTL4 showed substantial dispersion, and Bacteroides relative abundance was not transformed; these characteristics are acknowledged as limitations.

## Results

### Baseline characteristics of study participants

A total of 89 participants were enrolled (29 Con, 39 OB, 21 NAFLD). Baseline characteristics are presented in **Table 1**. The three groups were comparable in sex distribution (p = 0.219). NAFLD participants were older than controls (10.59 ± 1.48 vs. 9.19 ± 2.10 years, p = 0.036), and BMI, body fat percentage, and waist circumference increased progressively across groups (all p < 0.001).

**Table 1:** Baseline demographic, anthropometric, and biochemical characteristics.

Variable	Con (n=29)	OB (n=39)	NAFLD (n=21)	p value
Age, years	9.19 ± 2.10^a^	9.71 ± 1.86^ab^	10.59 ± 1.48^b^	0.036
Sex, male, n (%)	14 (48.3)	20 (51.3)	15 (71.4)	0.219
BMI, kg/m^2^	17.30 ± 3.45^a^	25.01 ± 3.10^b^	26.95 ± 2.84^c^	<0.001
BMI z-score	0.24 ± 1.19^a^	2.79 ± 0.66^b^	2.85 ± 0.50^b^	<0.001
Body fat, %	26.80 (25.40, 28.40)^a^	37.00 (35.35, 40.35)^b^	38.20 (36.60, 42.70)^b^	<0.001
Waist circumference, cm	59.26 ± 7.49^a^	75.02 ± 8.45^b^	82.83 ± 7.17^c^	<0.001
ALT, U/L	14.0 (12.0, 17.0)^a^	17.0 (13.0, 26.0)^a^	34.0 (24.0, 55.0)^b^	<0.001
AST, U/L	27.0 (23.0, 30.0)^ab^	24.0 (20.5, 28.5)^a^	32.0 (25.0, 45.0)^b^	0.006
TG, mmol/L	0.69 (0.60, 0.86)^a^	0.92 (0.76, 1.18)^b^	1.45 (1.01, 1.80)^c^	<0.001
HDL-C, mmol/L	1.72 ± 0.30^a^	1.45 ± 0.31^b^	1.29 ± 0.21^c^	<0.001
Fasting insulin, μIU/mL	9.77 (8.34, 12.39)^a^	12.90 (9.36, 21.30)^b^	22.30 (14.20, 31.90)^c^	<0.001
Leptin, pg/mL	564 (182, 949)^a^	2898 (1872, 4249)^b^	2948 (2387, 3783)^b^	<0.001
ANGPTL4, pg/mL	6180 ± 4740^a^	3068 ± 4795^b^	1514 ± 3297^b^	0.002
Adiponectin, ng/mL	8106 (6545, 10844)^a^	6150 (4668, 7179)^b^	5540 (4197, 6060)^b^	<0.001
IL-6, pg/mL	1.43 ± 0.82^a^	1.59 ± 0.87^ab^	2.10 ± 0.99^b^	0.037

Values: mean ± SD or median (Q1, Q3). Different superscript letters indicate statistically significant differences (p < 0.05) based on *post-hoc* pairwise comparisons: LSD test for normally distributed variables and Dunn-Bonferroni test for non-normally distributed variables. BMI, body mass index; ALT, alanine aminotransferase; AST, aspartate aminotransferase; TG, triglycerides; HDL-C, high-density lipoprotein cholesterol; ANGPTL4, angiopoietin-like 4; IL-6, interleukin-6.

NAFLD participants exhibited the most severe metabolic disturbances, with significantly elevated ALT (median: 34.0 vs. 17.0 vs. 14.0 U/L, p < 0.001), AST (32.0 vs. 24.0 vs. 27.0 U/L, p = 0.006), TG (1.45 vs. 0.92 vs. 0.69 mmol/L, p < 0.001), and fasting insulin (p < 0.001), and reduced HDL-C (1.29 ± 0.21 vs. 1.45 ± 0.31 vs. 1.72 ± 0.30 mmol/L, p < 0.001).

Leptin was similarly elevated in both obesity groups versus controls (p < 0.001), while ANGPTL4 and adiponectin were reduced (p = 0.002 and p < 0.001, respectively). IL-6 was elevated in NAFLD versus controls (2.10 ± 0.99 vs. 1.43 ± 0.82 pg/mL, p = 0.037).

### Gut microbiota alpha and beta diversity

Alpha diversity was assessed using Shannon and Chao1 indices. Chao1 differed significantly among groups (F = 4.98, p = 0.009), with the NAFLD group exhibiting the lowest species richness. Shannon diversity did not differ significantly among groups (F = 2.22, p = 0.115). Pairwise comparisons for Chao1 showed marginal differences between NAFLD and both Con and OB groups (FDR = 0.061 for both), while OB and Con did not differ (FDR = 0.976; **Table 2**).

**Table 2:** Alpha diversity indices across groups.

Index	Con (n=29)	OB (n=39)	NAFLD (n=21)	F	p value
Shannon	2.24 ± 0.29	2.17 ± 0.42	1.98 ± 0.58	2.22	0.115
Chao1	31.8 ± 4.01	31.8 ± 3.17	28.3 ± 6.79	4.98	0.009

Values: Mean ± SD. Welch’s ANOVA with *post-hoc* pairwise comparisons (Benjamini-Hochberg FDR correction).

Chao1 pairwise comparisons: OB vs NAFLD FDR = 0.061; Con vs NAFLD FDR = 0.061; OB vs Con FDR = 0.976. Shannon pairwise comparisons all non-significant (FDR > 0.05).

Beta diversity (Bray-Curtis PCoA; **Figure 1**) differed significantly among groups (PERMANOVA: F = 2.87, R^2^ = 0.063, p = 0.002). Pairwise PERMANOVA revealed significant separation between NAFLD and Con (F = 4.55, R^2^ = 0.087, p = 0.002, FDR = 0.006) and between NAFLD and OB (F = 2.62, R^2^ = 0.043, p = 0.013, FDR = 0.020), while OB and Con did not differ significantly (F = 1.80, R^2^ = 0.027, p = 0.070, FDR = 0.070). PERMDISP analysis indicated significant heterogeneity in multivariate dispersion among groups (F = 6.62, p = 0.002).

**Figure 1:**
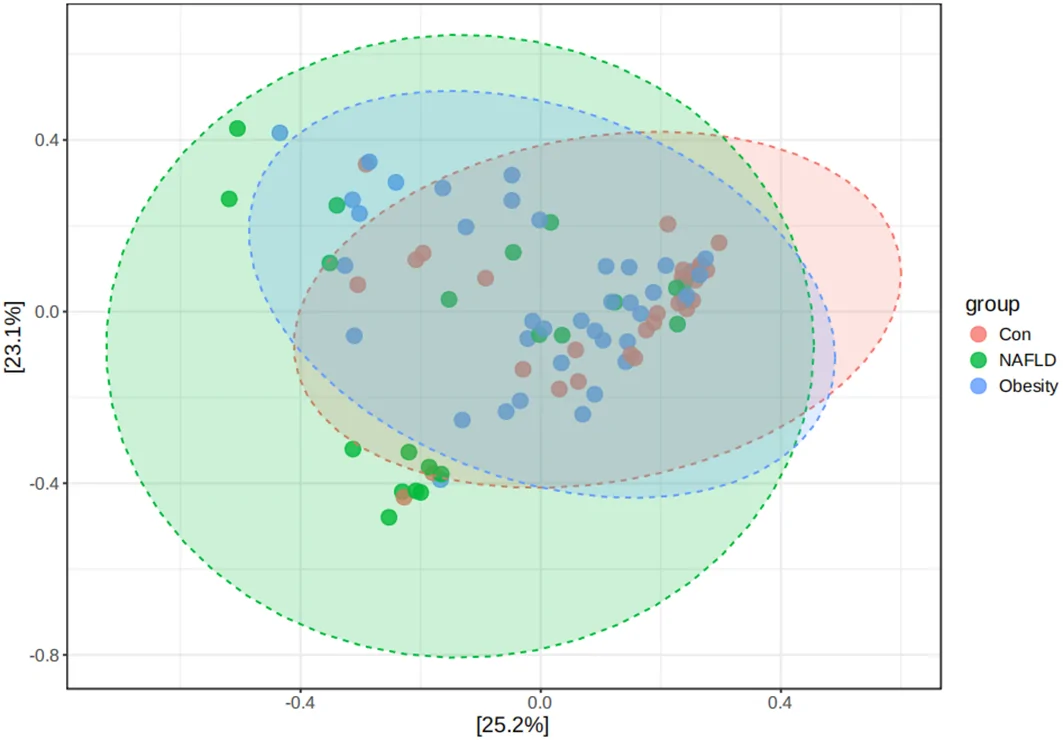
Principal coordinate analysis (PCoA) of gut microbiota based on Bray-Curtis distance. Each point represents a sample. Con (n = 29, red), OB (n = 39, blue), and NAFLD (n = 21, green). Ellipses represent 95% confidence regions. PCoA1 and PCoA2 explain 25.2% and 23.1% of the total variance, respectively. PERMANOVA: F = 2.87, R^2^ = 0.063, p = 0.002 (999 permutations). Pairwise PERMANOVA: NAFLD vs Con FDR = 0.006; NAFLD vs OB FDR = 0.020; OB vs Con FDR = 0.070. PERMDISP: F = 6.62, p = 0.002.

### Taxonomic differences in gut microbiota composition

LEfSe identified 13 differentially abundant genera (LDA > 2.0; **Supplementary Figure S1**; **Supplementary Table S1**). Healthy controls were enriched in Bacteroides (LDA = 4.89), Alistipes (LDA = 4.01), Oscillospira (LDA = 3.70), and Parabacteroides (LDA = 3.64). The OB group was characterized by Faecalibacterium (LDA = 4.55), Prevotella (LDA = 4.38), Roseburia (LDA = 4.09), Phascolarctobacterium (LDA = 3.90), and Gemmiger (LDA = 3.79). NAFLD children exhibited enrichment of Megamonas (LDA = 4.63), Blautia (LDA = 4.09), Coprococcus (LDA = 3.61), and Dorea (LDA = 3.53). Boxplots of representative genera are shown in **Figure 2**. Only Bacteroides remained significant after FDR correction (q = 0.014); the other genera showed nominal significance only.

**Figure 2:**
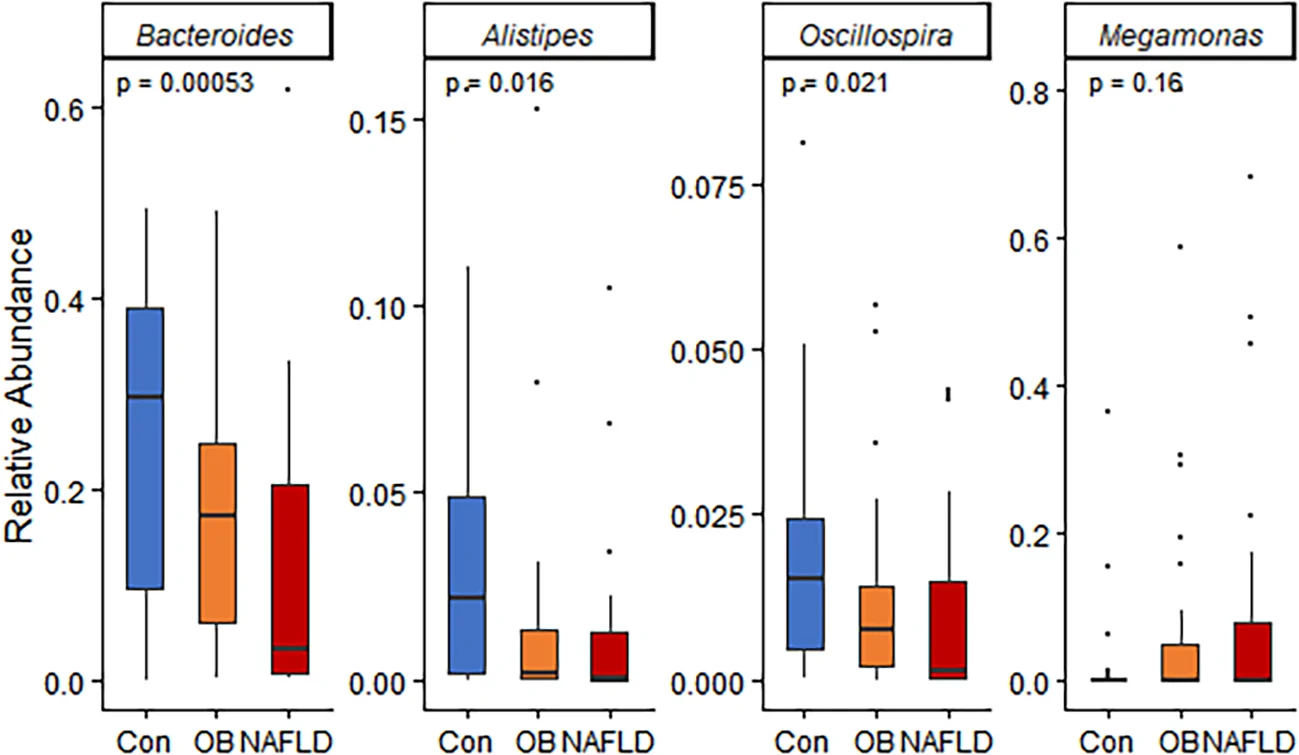
Boxplots of representative differentially abundant genera. Boxplots showing relative abundance of **(A)** Bacteroides, **(B)** Alistipes, **(C)** Oscillospira, and **(D)** Megamonas across Con (blue, n = 29), OB (orange, n = 39), and NAFLD (red, n = 21) groups. Boxes represent median and IQR; whiskers extend to 1.5 × IQR. P values from Kruskal-Wallis test.

ANCOM-BC2 with adjustment for age, sex, and BF% identified several genera after FDR correction. In NAFLD vs Con, Megamonas (log2FC = 3.54, q = 0.005) and Veillonella (log2FC = 2.46, q = 0.031) were significantly enriched in NAFLD. In OB vs NAFLD, Megamonas (log2FC = −2.77, q = 0.013) and Megasphaera (log2FC = 3.21, q = 0.008) differed significantly. In OB vs Con, Prevotella (log2FC = −2.40, q = 0.015) and Haemophilus (log2FC = 2.17, q = 0.015) were significantly different. Bacteroides showed a consistent direction of effect (NAFLD vs Con: log2FC = −1.36, p = 0.126; OB vs NAFLD: log2FC = 1.57, p = 0.063) but did not reach FDR significance after covariate adjustment.

Direct comparison between OB and NAFLD groups using Mann-Whitney U tests and logistic regression adjusting for age, sex, BMI, and Tanner stage showed no genus significantly distinguished OB from NAFLD after adjustment (all p > 0.05). At the phylum level, the gut microbiota was dominated by Firmicutes, Bacteroidetes, Proteobacteria, and Actinobacteria across all three groups (**Supplementary Figure S2**). Genus-level composition is shown in **Supplementary Figure S3**.

### Functional prediction

PICRUSt2 predicted lower abundance of nine SCFA-related pathways in NAFLD/OB groups compared with controls (all p < 0.05 after FDR correction; **Figure 3**; **Supplementary Table S2**). These included glycolysis I (p = 0.0019, q = 0.013), homolactic fermentation (p = 0.0036, q = 0.013), hexitol fermentation (p = 0.0037, q = 0.013), glycolysis II (p = 0.0038, q = 0.013), L-lysine fermentation to acetate and butanoate (p = 0.0062, q = 0.017), pyruvate fermentation to acetate and lactate II (p = 0.0081, q = 0.019), acetyl-CoA fermentation to butanoate II (p = 0.010, q = 0.020), mixed acid fermentation (p = 0.014, q = 0.024), and pyruvate fermentation to propanoate I (p = 0.028, q = 0.044). Pairwise comparisons showed that all significant differences were between the obesity groups (OB or NAFLD) and controls; no significant differences were detected between NAFLD and OB groups for any pathway.

**Figure 3:**
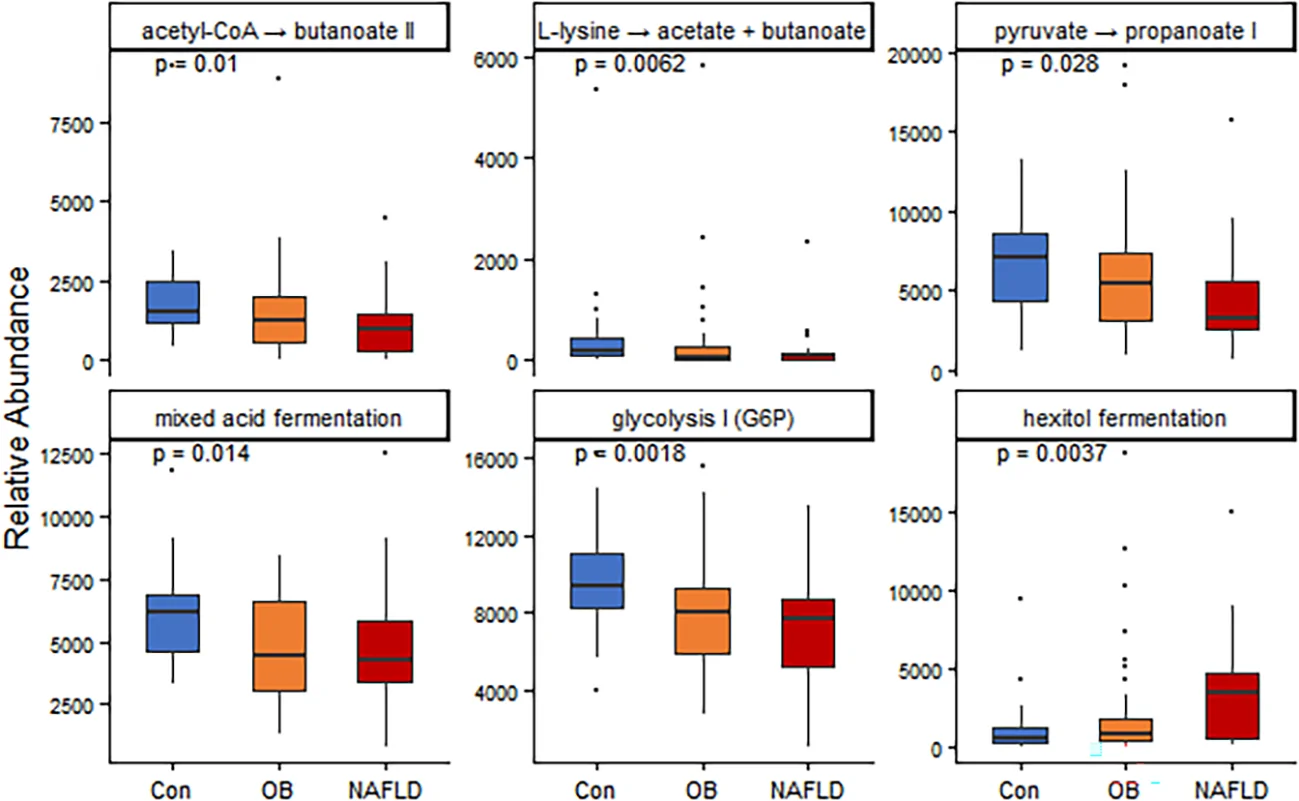
PICRUSt2-predicted abundance of SCFA-related MetaCyc pathways. Boxplots showing predicted relative abundances of six representative SCFA biosynthetic pathways: **(A)** acetyl-CoA fermentation to butanoate II, **(B)** L-lysine fermentation to acetate and butanoate, **(C)** pyruvate fermentation to propanoate I, **(D)** mixed acid fermentation, **(E)** glycolysis I (from glucose 6-phosphate), and **(F)** hexitol fermentation to lactate, formate, ethanol and acetate. Con (blue, n = 29), OB (orange, n = 39), NAFLD (red, n = 21). Boxes represent median and IQR; whiskers extend to 1.5 × IQR. **Global differences among groups were tested by Kruskal-Wallis with Benjamini-Hochberg FDR correction across the 14 prespecified pathways. Pairwise comparisons used Dunn’s post-hoc test with Bonferroni correction. *q < 0.05, **q < 0.01, ***q < 0.001.

### Correlation between gut microbiota and metabolic markers

Spearman correlation (**Figure 4**; **Supplementary Table S3**) revealed that Bacteroides was negatively associated with leptin (r = −0.341) and ALT (r = −0.287), and positively associated with ANGPTL4 (r = 0.460). After Benjamini-Hochberg FDR correction across 30 tests, five associations remained significant: Bacteroides-ANGPTL4 (q < 0.001), Bacteroides-leptin (q = 0.012), Bacteroides-ALT (q = 0.027), Oscillospira-ALT (q = 0.045), and Parabacteroides-ALT (q = 0.012).

**Figure 4:**
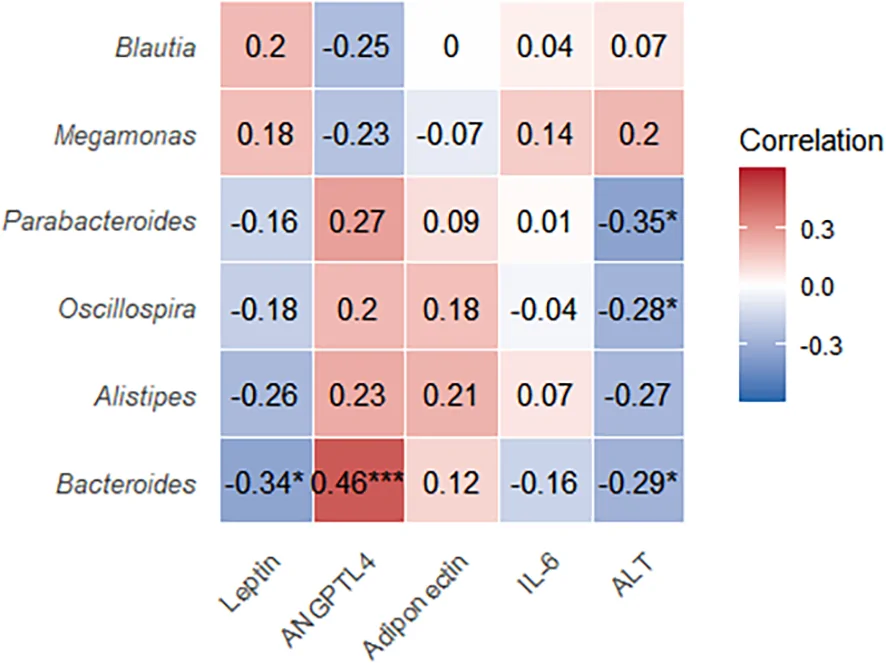
Spearman correlation heatmap between differentially abundant genera and serum metabolic markers. Heatmap showing Spearman rank correlation coefficients between six LEfSe-identified genera (Bacteroides, Alistipes, Oscillospira, Parabacteroides, Megamonas, Blautia) and five serum markers (Leptin, ANGPTL4, Adiponectin, IL-6, ALT). Blue indicates negative correlation; red indicates positive correlation. *q < 0.05, ***q < 0.001 after Benjamini-Hochberg FDR correction across 30 tests. Five associations survived correction: Bacteroides-ANGPTL4 (q < 0.001), Bacteroides-leptin (q = 0.012), Bacteroides-ALT (q = 0.027), Oscillospira-ALT (q = 0.045), and Parabacteroides-ALT (q = 0.012).

Partial correlation controlling for age and BMI attenuated the Bacteroides-leptin association (r = −0.159, p = 0.145), while the Bacteroides-ANGPTL4 association remained significant (r = 0.251, p = 0.021).

Hierarchical regression was performed with four models (**Table 3**). In Model 1 (age + BMI), BMI was significantly associated with ANGPTL4 (β = −0.298, p = 0.012). After adding sex and Tanner stage in Model 2, the model explained 17.8% of ANGPTL4 variance. Addition of Bacteroides in Model 3 significantly improved the model (ΔR^2^ = 0.057, p = 0.016), with Bacteroides independently associated with ANGPTL4 (β = 0.254, p = 0.016). After further adjustment for group status in Model 4, the association was attenuated and no longer reached statistical significance (β = 0.204, p = 0.058; **Table 3**). Sensitivity analysis excluding one participant with extreme ALT values (n = 84) yielded consistent results.

**Table 3:** Hierarchical multiple linear regression: Bacteroides abundance and serum ANGPTL4.

Variable	Model 1 β (p)	Model 2 β (p)	Model 3 β (p)	Model 4 β (p)
Age	-0.105 (0.369)	-0.290 (0.071)	-0.373 (0.020)	-0.415 (0.011)
BMI	-0.298 (0.012)	-0.193 (0.124)	-0.102 (0.420)	0.205 (0.318)
Sex	—	0.218 (0.363)	0.156 (0.505)	0.322 (0.197)
Tanner	—	0.211 (0.169)	0.249 (0.098)	0.198 (0.189)
Bacteroides	—	—	0.254 (0.016)	0.204 (0.058)
Group (OB vs NAFLD)	—	—	—	0.114 (0.664)
Group (Con vs NAFLD)	—	—	—	0.793 (0.075)
R^2^	0.130	0.178	0.235	0.268
ΔR^2^	—	0.048	0.057 (p=0.016)	0.034 (p=0.168)

Model 1: age + BMI. Model 2: Model 1 + sex + Tanner. Model 3: Model 2 + Bacteroides. Model 4: Model 3 + group. β, standardized coefficient. Group was dummy-coded using NAFLD as reference group. Two dummy variables were entered: OB versus NAFLD, Con versus NAFLD.

Sensitivity analysis using BMI z-score ≥ 2 to define obesity (instead of BF% ≥ 30%) yielded consistent results for the Bacteroides-ANGPTL4 association (partial r = 0.253, p = 0.019, n = 87).

## Discussion

In this study, we characterized the gut microbiota of Chinese children with obesity and NAFLD and identified key taxonomic and functional alterations associated with disease status. Our principal findings are: (1) NAFLD children exhibited significantly reduced species richness (Chao1) and altered beta diversity; (2) LEfSe identified enrichment of SCFA-producing genera (Bacteroides, Alistipes, Oscillospira, Parabacteroides) in healthy controls, with depletion of these genera in NAFLD, and enrichment of Megamonas, Blautia, Coprococcus, and Dorea in NAFLD; (3) SCFA-related pathways were predicted to be lower in abundance in NAFLD; and (4) Bacteroides abundance was independently associated with serum ANGPTL4 after adjustment for age, BMI, sex, and Tanner stage (Model 3), although this association was attenuated after additional adjustment for group status (Model 4).

The significant reduction in species richness and altered beta diversity align with the recent meta-analysis by Zoggeler et al. (7). Our PERMANOVA result, with significant pairwise separation between NAFLD and both Con and OB, indicates differences in community composition. PERMDISP indicated significant heterogeneity in multivariate dispersion, suggesting that part of the PERMANOVA signal may be attributable to unequal within-group dispersion, and this should be interpreted with caution. The modest effect size indicates that only a small proportion of gut microbial community variation was explained by obesity and NAFLD status, consistent with the multifactorial nature of gut microbiota composition, which is influenced by diet, genetics, and environmental factors beyond liver fat accumulation.

The enrichment of SCFA-producing genera in healthy controls is a consistent finding. Bacteroides, Alistipes, Oscillospira, and Parabacteroides are well-characterized SCFA producers (31). Parabacteroides has been associated with improved metabolic profiles and reduced hepatic steatosis in an exercise model of early obesity (31). A recent review confirmed the protective role of Bacteroides in NAFLD through AMPK pathway activation, reduction of hepatic inflammation, and improvement of insulin resistance (16). In contrast, Megamonas enrichment in NAFLD corroborates the findings of Zhou et al., who demonstrated through metagenomic analysis that Megamonas hypermegale and Megamonas rupellensis were significantly enriched in Chinese children with NAFLD (30). However, our 16S rRNA data cannot resolve Megamonas to the species level, and genus-level assignment should be interpreted with this limitation. The consistency of Megamonas enrichment across independent Chinese pediatric cohorts suggests it may represent a population-specific microbial signature of pediatric NAFLD.

Direct comparison between OB and NAFLD groups showed that no genus significantly distinguished the two groups after adjustment, indicating that the microbial differences between these groups are largely explained by age, adiposity, and pubertal development. This is further supported by pairwise PERMANOVA, which revealed significant separation between NAFLD and Con and between NAFLD and OB, while OB and Con did not differ significantly, suggesting a gradient of microbial alteration across the obesity-NAFLD spectrum.

It should be noted that microbial metabolic function is species- and strain-dependent, and 16S genus-level resolution cannot establish the metabolic activity of the organisms present. Genera enriched in the NAFLD group may themselves contain SCFA-producing organisms. The binary classification of taxa as “beneficial SCFA producers” versus “adverse bacteria” is therefore an oversimplification, and the functional data are themselves predicted rather than measured.

The PICRUSt2-predicted lower abundance of SCFA-related pathways is consistent with the taxonomic findings, though these results represent predicted functional potential rather than direct measurements of pathway activity or SCFA concentrations. SCFAs, particularly butyrate, activate AMPK and PPAR-gamma, promoting fatty acid oxidation while inhibiting *de novo* lipogenesis (9, 10). The predicted functional changes combined with taxonomic depletion of SCFA producers provide consistent, though predicted, evidence for altered SCFA-mediated metabolic regulation in NAFLD (8, 11). Notably, pairwise comparisons showed that all significant differences were between the obesity groups and controls, with no significant differences between NAFLD and OB groups for any pathway, suggesting that impaired SCFA biosynthetic potential is more closely tied to obesity itself than to NAFLD status.

ANCOM-BC2 with adjustment for age, sex, and BF% confirmed Megamonas enrichment in NAFLD, consistent with the LEfSe findings. Veillonella, Megasphaera, Prevotella, and Haemophilus also differed significantly in specific pairwise comparisons. Bacteroides showed a consistent direction of effect but did not reach FDR significance after covariate adjustment. These results support the biological plausibility of the taxonomic findings, although the NAFLD-specific taxonomic signature was less pronounced than the obesity-related signature.

A notable finding is the independent positive association between Bacteroides abundance and serum ANGPTL4. To our knowledge, this is the first study to report this association in pediatric NAFLD. In hierarchical regression, Bacteroides contributed an additional 5.7% of ANGPTL4 variance beyond age, BMI, sex, and Tanner stage. However, after further adjustment for group status, the association was attenuated and no longer reached statistical significance, suggesting that part of the association is explained by disease status. Given the modest effect size and the cross-sectional design, this association should be characterized as exploratory and requiring independent replication rather than as evidence of a defined microbiota-ANGPTL4 axis.

The initial negative Bacteroides-leptin correlation was attenuated after adjustment for age and BMI, underscoring the importance of adiposity adjustment. Bacteroides-ALT, Oscillospira-ALT, and Parabacteroides-ALT associations also survived FDR correction but were not further examined after adiposity adjustment. Adiponectin reductions in obesity groups are consistent with established literature on adipokine dysregulation in obesity and NAFLD (22).

Mechanistically, ANGPTL4 functions as a fasting- and lipid-induced LPL inhibitor (13), and gut microbiota colonization suppresses its intestinal expression (14). However, ANGPTL4 is a circulating protein produced by multiple tissues, including adipose tissue, liver, and intestine. While our findings demonstrate an association between gut Bacteroides abundance and serum ANGPTL4, the relative contribution of intestinal versus hepatic and adipose ANGPTL4 to circulating levels cannot be determined from our data. The microbiota-ANGPTL4 relationship is likely bidirectional and tissue-specific, and future studies using tissue-specific knockout models or intestinal ANGPTL4 measurements would be needed to establish the direction and source of this association.

BF% was used rather than BMI because it directly measures adiposity and BMI tends to under-detect obesity in children (32, 33). This approach is supported by pediatric body fat references (20, 21), and the ≥30% threshold is consistent with the reference curves of McCarthy et al. (32). Sensitivity analysis using BMI z-score ≥ 2 instead of BF% ≥ 30% yielded consistent results for the Bacteroides-ANGPTL4 association. We acknowledge that a fixed BF% threshold does not account for age- and sex-related differences in body composition.

### Limitations

Several limitations should be acknowledged. First, the cross-sectional design precludes causal inference. Second, NAFLD was diagnosed by ultrasonography rather than biopsy or MRS, and steatosis severity was not graded. Third, the small NAFLD sample (n = 21) limits statistical power, and ANCOM-BC2 with covariate adjustment identified fewer FDR-significant genera than LEfSe. Fourth, 16S rRNA sequencing provides only genus-level resolution and cannot distinguish strains differing in SCFA metabolic capacity; PICRUSt2 predictions are hypothesis-generating, and direct SCFA measurements were not performed. Serum ANGPTL4 reflects circulating rather than intestinal protein levels, and its tissue of origin cannot be determined. Technical replicates were not performed, and regression diagnostics indicated substantial dispersion of ANGPTL4 with untransformed Bacteroides abundance. Finally, dietary intake, physical activity, pubertal stage, socioeconomic factors, and probiotic/prebiotic use were not systematically recorded, and the single-center design and modest PERMANOVA effect size limit generalizability.

## Conclusion

In conclusion, Chinese children with obesity and NAFLD exhibit significant gut microbial dysbiosis characterized by reduced species richness (Chao1), depletion of SCFA-producing genera, enrichment of Megamonas, and lower predicted abundance of SCFA-related pathways. The independent Bacteroides-ANGPTL4 association suggests a potential microbiota-host metabolic link that warrants prospective evaluation in pediatric NAFLD.

## Data Availability

The datasets presented in this study can be found in online repositories. The names of the repository/repositories and accession number(s) can be found in the article/**Supplementary Material**.

## References

[1] Rinella ME Lazarus JV Ratziu V Francque SM Sanyal AJ Kanwal F . A multisociety Delphi consensus statement on new fatty liver disease nomenclature. Hepatology. (2023) 78:1966–86. doi: 10.1097/HEP.0000000000000520 37363821 PMC10653297

[2] Lee EJ Choi M Ahn SB Yoo JJ Kang SH Cho Y . Prevalence of nonalcoholic fatty liver disease in pediatrics and adolescents: a systematic review and meta-analysis. World J Pediatr. (2024) 20:569–80. doi: 10.1007/s12519-024-00814-1 38771552

[3] Younossi ZM Golabi P Paik JM Henry A Van Dongen C Henry L . The global epidemiology of nonalcoholic fatty liver disease (NAFLD) and nonalcoholic steatohepatitis (NASH): a systematic review. Hepatology. (2023) 77:1335–47. doi: 10.1097/HEP.0000000000000004 36626630 PMC10026948

[4] Schwimmer JB Thai NQN Noon SL Ugalde-Nicalo P Anderson SR Chun LF . Long-term mortality and extrahepatic outcomes in 1096 children with MASLD: a retrospective cohort study. Hepatology. (2026) 83:561–74. doi: 10.1097/HEP.0000000000001357 40262118 PMC12353144

[5] Tilg H Adolph TE Trauner M . Gut-liver axis: Pathophysiological concepts and clinical implications. Cell Metab. (2022) 34:1700–18. doi: 10.1016/j.cmet.2022.09.017 36208625

[6] Du L Zhang K Liang L Yang Y Lu D Zhou Y . Multi-omics analyses of the gut microbiota and metabolites in children with metabolic dysfunction-associated steatotic liver disease. mSystems. (2025) 10:e0114824. doi: 10.1128/msystems.01148-24 40084870 PMC12013275

[7] Zöggeler T Kavallar AM Pollio AR Aldrian D Decristoforo C Scholl-Bürgi S . Meta-analysis of shotgun sequencing of gut microbiota in obese children with MASLD or MASH. Gut Microbes. (2025) 17:2508951. doi: 10.1080/19490976.2025.2508951 40396204 PMC12101585

[8] Jiang L Du LD Zeng J Chu HK Peng Z Fan JG . Pediatric metabolic dysfunction-associated steatotic liver disease and the gut microbiome: from research landscape to targeted modulation. Clin Mol Hepatol. (2026) 32:53–68. doi: 10.3350/cmh.2025.0718 40825719 PMC12835764

[9] Canfora EE Jocken JW Blaak EE . Short-chain fatty acids in control of body weight and insulin sensitivity. Nat Rev Endocrinol. (2015) 11:577–91. doi: 10.1038/nrendo.2015.128 26260141

[10] den Besten G van Eunen K Groen AK Venema K Reijngoud DJ Bakker BM . The role of short-chain fatty acids in the interplay between diet, gut microbiota, and host energy metabolism. J Lipid Res. (2013) 54:2325–40. doi: 10.1194/jlr.R036012 23821742 PMC3735932

[11] Boursier J Mueller O Barret M Machado M Fizanne L Araujo-Perez F . The severity of nonalcoholic fatty liver disease is associated with gut dysbiosis and shift in the metabolic function of the gut microbiota. Hepatology. (2016) 63:764–75. doi: 10.1002/hep.28356 26600078 PMC4975935

[12] Loomba R Seguritan V Li W Long T Klitgord N Bhatt A . Gut microbiome-based metagenomic signature for non-invasive detection of advanced fibrosis in human nonalcoholic fatty liver disease. Cell Metab. (2017) 25:1054–1062.e5. doi: 10.1016/j.cmet.2017.04.001 28467925 PMC5502730

[13] Kersten S . Role and mechanism of the action of angiopoietin-like protein ANGPTL4 in plasma lipid metabolism. J Lipid Res. (2021) 62:100150. doi: 10.1016/j.jlr.2021.100150 34801488 PMC8666355

[14] Bäckhed F Ding H Wang T Hooper LV Koh GY Nagy A . The gut microbiota as an environmental factor that regulates fat storage. Proc Natl Acad Sci USA. (2004) 101:15718–23. doi: 10.1073/pnas.0407076101 15505215 PMC524219

[15] Yuan X Chen R Zhang Y Lin X Yang X McCormick KL . Gut microbiota of Chinese obese children and adolescents with and without insulin resistance. Front Endocrinol (Lausanne). (2021) 12:636272. doi: 10.3389/fendo.2021.636272 33815293 PMC8018175

[16] Zhang J Zhou J He Z Li H . Bacteroides and NAFLD: pathophysiology and therapy. Front Microbiol. (2024) 15:1288856. doi: 10.3389/fmicb.2024.1288856 38572244 PMC10988783

[17] Yuan X Zhang Y Lin X Yang X Chen R . Association of gut microbiota and glucose metabolism in children with disparate degrees of adiposity. Pediatr Obes. (2023) 18:e13009. doi: 10.1111/ijpo.13009 36704910

[18] Yuan X Chen R Zhang Y Lin X Yang X . Altered gut microbiota in children with hyperuricemia. Front Endocrinol (Lausanne). (2022) 13:848715. doi: 10.3389/fendo.2022.848715 35574004 PMC9091909

[19] Yuan X Chen R McCormick KL Zhang Y Lin X Yang X . The role of the gut microbiota on the metabolic status of obese children. Microb Cell Fact. (2021) 20:53. doi: 10.1186/s12934-021-01548-9 33639944 PMC7916301

[20] Lee K Lee S Kim SY Kim SJ Kim YJ . Percent body fat cutoff values for classifying overweight and obesity recommended by the International Obesity Task Force (IOTF) in Korean children. Asia Pac J Clin Nutr. (2007) 16:649–55 18042524

[21] Guo B Wu Q Gong J Xiao Z Tang Y Shang J . Gender difference in body fat for healthy Chinese children and adolescents. Child Obes. (2016) 12:144–54. doi: 10.1089/chi.2015.0139 26974254

[22] Goldner D Lavine JE . Nonalcoholic fatty liver disease in children: unique considerations and challenges. Gastroenterology. (2020) 158:1967–1983.e1. doi: 10.1053/j.gastro.2020.01.048 32201176

[23] Li H Zong XN Ji CY Mi J . Body mass index cut-offs for overweight and obesity in Chinese children and adolescents aged 2-18 years. Zhonghua Liu Xing Bing Xue Za Zhi. (2010) 31:616–20 21163089. Chinese.

[24] Bolyen E Rideout JR Dillon MR Bokulich NA Abnet CC Al-Ghalith GA . Reproducible, interactive, scalable and extensible microbiome data science using QIIME 2. Nat Biotechnol. (2019) 37:852–7. doi: 10.1038/s41587-019-0209-9 31341288 PMC7015180

[25] Callahan BJ McMurdie PJ Rosen MJ Han AW Johnson AJ Holmes SP . DADA2: high-resolution sample inference from Illumina amplicon data. Nat Methods. (2016) 13:581–3. doi: 10.1038/nmeth.3869 27214047 PMC4927377

[26] DeSantis TZ Hugenholtz P Larsen N Rojas M Brodie EL Keller K . Greengenes, a chimera-checked 16S rRNA gene database and workbench compatible with ARB. Appl Environ Microbiol. (2006) 72:5069–72. doi: 10.1128/aem.03006-05 16820507 PMC1489311

[27] Chong J Liu P Zhou G Xia J . Using MicrobiomeAnalyst for comprehensive statistical, functional, and meta-analysis of microbiome data. Nat Protoc. (2020) 15:799–821. doi: 10.1038/s41596-019-0264-1 31942082

[28] Segata N Izard J Waldron L Gevers D Miropolsky L Garrett WS . Metagenomic biomarker discovery and explanation. Genome Biol. (2011) 12:R60. doi: 10.1186/gb-2011-12-6-r60 21702898 PMC3218848

[29] Douglas GM Maffei VJ Zaneveld JR Yurgel SN Brown JR Taylor CM . PICRUSt2 for prediction of metagenome functions. Nat Biotechnol. (2020) 38:685–8. doi: 10.1038/s41587-020-0548-6 32483366 PMC7365738

[30] Zhou J Zhang Q Zhao Y Zou Y Chen M Zhou S . The relationship of Megamonas species with nonalcoholic fatty liver disease in children and adolescents revealed by metagenomics of gut microbiota. Sci Rep. (2022) 12:22001. doi: 10.1038/s41598-022-25140-2 36539432 PMC9767906

[31] Carbajo-Pescador S Porras D García-Mediavilla MV Martínez-Flórez S Juarez-Fernández M Cuevas MJ . Beneficial effects of exercise on gut microbiota functionality and barrier integrity, and gut-liver crosstalk in an *in vivo* model of early obesity and non-alcoholic fatty liver disease. Dis Model Mech. (2019) 12:dmm039206. doi: 10.1242/dmm.039206 30971408 PMC6550047

[32] McCarthy HD Cole TJ Fry T Jebb SA Prentice AM . Body fat reference curves for children. Int J Obes (Lond). (2006) 30:598–602. doi: 10.1038/sj.ijo.0803232 16570089

[33] Laurson KR Eisenmann JC Welk GJ . BMI standards based on agreement with health-related body fat. Am J Prev Med. (2011) 41:S100–5. doi: 10.1016/j.amepre.2011.07.004 21961608

