# Supplementary material for: Gut microbiota dysbiosis and altered short-chain fatty acid biosynthetic pathways in Chinese children with obesity and nonalcoholic fatty liver disease
Source: Front Endocrinol (Lausanne). 2026 Oct 30;17:1965476. doi: 10.3389/fendo.2026.1965476 (PMC13759286; doi:10.3389/fendo.2026.1965476)
Supplement: Supplementary file 1 [file SupplementaryFile1.docx]

**Supplementary Methods. ELISA assay performance characteristics.**

Serum leptin was measured using a commercial ELISA kit (Abcam, Cambridge, UK), with inter-assay and intra-assay coefficients of variation (CV) of 2.4% and 2.7%, respectively. Serum ANGPTL4 was measured using a commercial ELISA kit (ab99974, Abcam, Cambridge, UK), with inter-assay and intra-assay CV < 10% and < 8%, respectively, and a detection range of 27.4–20,000 pg/mL (sensitivity < 20 pg/mL). Serum IL-6 was measured using a commercial ELISA kit (Abcam, Cambridge, UK), with inter-assay CV of 4.4%.

**Table S1. LEfSe-identified differentially abundant genera**

| **Group** | **Genus** | **LDA** | **p** | **FDR** |
| --- | --- | --- | --- | --- |
| **Con** | Bacteroides | 4.89 | 0.00050 | **0.014** |
|  | Alistipes | 4.01 | 0.017 | 0.158 |
|  | Oscillospira | 3.70 | 0.020 | 0.158 |
|  | Parabacteroides | 3.64 | 0.151 | 0.336 |
| **OB** | Faecalibacterium | 4.55 | 0.067 | 0.325 |
|  | Prevotella | 4.38 | 0.148 | 0.336 |
|  | Roseburia | 4.09 | 0.186 | 0.360 |
|  | Phascolarctobacterium | 3.90 | 0.102 | 0.325 |
|  | Gemmiger | 3.79 | 0.087 | 0.325 |
| **NAFLD** | Megamonas | 4.63 | 0.152 | 0.360 |
|  | Blautia | 4.09 | 0.170 | 0.360 |
|  | Coprococcus | 3.61 | 0.817 | 0.918 |
|  | Dorea | 3.53 | 0.200 | 0.365 |

**Note**: LDA > 2.0. p values from Kruskal-Wallis test. Only Bacteroides remained significant after FDR correction.

**Table S2. PICRUSt2-predicted MetaCyc pathway abundances across groups**

| **MetaCyc Pathway** | **Con (n=29)** | **OB (n=39)** | **NAFLD (n=21)** | **p** | **q** |
| --- | --- | --- | --- | --- | --- |
| glycolysis I (G6P) | 9357.5 (8246.7, 11077.6) | 8047.3 (5869.1, 9284.2) | 7695.6 (5174.8, 8720.0) | 0.0019 | **0.013** |
| homolactic fermentation | 8161.9 (7088.5, 9668.9) | 7052.5 (4999.2, 7875.3) | 6666.2 (4121.5, 7471.3) | 0.0036 | **0.013** |
| hexitol fermentation | 584.6 (375.0, 1335.9) | 916.5 (528.2, 1920.3) | 3469.8 (673.3, 4825.5) | 0.0037 | **0.013** |
| glycolysis II (F6P) | 8073.0 (6579.7, 9976.5) | 6383.3 (4672.9, 7919.4) | 5649.8 (4141.7, 6595.5) | 0.0038 | **0.013** |
| L-lysine → acetate + butanoate | 167.8 (85.7, 445.4) | 57.6 (19.5, 238.9) | 69.6 (11.6, 124.8) | 0.0062 | **0.017** |
| pyruvate → acetate + lactate II | 10077.3 (9754.9, 11510.0) | 9426.6 (7895.4, 10491.1) | 8346.8 (5629.9, 10185.9) | 0.0081 | **0.019** |
| acetyl-CoA → butanoate II | 1521.7 (1193.0, 2469.1) | 1272.3 (531.6, 1992.3) | 937.9 (257.5, 1461.7) | 0.010 | **0.020** |
| mixed acid fermentation | 6187.8 (4639.9, 6871.5) | 4453.9 (3009.7, 6583.4) | 4287.8 (3408.3, 5820.3) | 0.014 | **0.024** |
| pyruvate → propanoate I | 7151.6 (4369.4, 8604.9) | 5520.2 (3176.7, 7342.3) | 3313.6 (2555.7, 5632.3) | 0.028 | **0.044** |
| succinate → butanoate | 272.1 (177.8, 493.6) | 205.3 (130.9, 343.6) | 196.3 (71.6, 303.2) | 0.060 | 0.085 |
| heterolactic fermentation | 1030.4 (372.9, 1403.8) | 475.1 (169.0, 933.1) | 597.9 (120.9, 1262.4) | 0.075 | 0.095 |
| TCA cycle IV | 296.0 (72.8, 946.9) | 658.6 (124.1, 1508.1) | 942.0 (68.9, 2531.1) | 0.296 | 0.345 |
| pyruvate → butanoate | 691.6 (202.6, 1282.5) | 609.9 (149.2, 1178.1) | 331.9 (105.5, 820.7) | 0.471 | 0.507 |
| fatty acid salvage | 0 (0, 0) | 0 (0, 0) | 0 (0, 0) | 0.825 | 0.825 |

**Note**: Values are median (Q1, Q3). Kruskal-Wallis with Benjamini-Hochberg FDR correction across 14 pathways. Pairwise comparisons (Dunn test with Bonferroni correction) are provided in Supplementary Text.

**Table S3. Spearman correlation matrix between genera and serum metabolic markers**

| Genus | Leptin | ANGPTL4 | Adiponectin | IL-6 | ALT |
| --- | --- | --- | --- | --- | --- |
| Bacteroides | −0.341 | **0.460** | 0.117 | −0.163 | −0.287 |
| Alistipes | −0.261 | 0.235 | 0.213 | 0.075 | −0.267 |
| Oscillospira | −0.181 | 0.199 | 0.178 | −0.035 | −0.285 |
| Parabacteroides | −0.161 | 0.270 | 0.093 | 0.014 | −0.348 |
| Megamonas | 0.181 | −0.227 | −0.073 | 0.140 | 0.203 |
| Blautia | 0.196 | −0.249 | −0.004 | 0.042 | 0.073 |

**Note**: **Bold** indicates q < 0.05 after Benjamini-Hochberg FDR correction across 30 tests. Five associations survived correction: Bacteroides-ANGPTL4 (q < 0.001), Bacteroides-leptin (q = 0.012), Bacteroides-ALT (q = 0.027), Oscillospira-ALT (q = 0.045), Parabacteroides-ALT (q = 0.012).

**Figure S1. Linear discriminant analysis Effect Size (LEfSe) at the genus level.**

Bar plot showing the top 15 genera with the highest LDA scores (LDA > 2.0). Colors indicate the group in which each genus is enriched: Con (red), NAFLD (blue), and Obesity (green).

**Figure S2. Phylum-level taxonomic composition across groups.**

Stacked bar plot showing the relative abundance of dominant phyla (Firmicutes, Bacteroidetes, Proteobacteria, and Actinobacteria) in Con (n = 29), OB (n = 39), and NAFLD (n = 21) groups.

### **Figure S3. Gut microbiota composition at genus levels.**

Stacked bar plot of genus-level relative abundance (top 10 genera) across groups. Con (n=29), OB (n=39), NAFLD (n=21).
